# Effectiveness of a Hydrophilic Curcumin-Based Formulation in Coadjuvating the Therapeutic Effect of Intravitreal Dexamethasone in Subjects With Diabetic Macular Edema

**DOI:** 10.3389/fphar.2021.726104

**Published:** 2022-01-04

**Authors:** Mariacristina Parravano, Davide Allegrini, Adriano Carnevali, Eliana Costanzo, Giuseppe Giannaccare, Paola Giorno, Vincenzo Scorcia, Giorgio Alfredo Spedicato, Monica Varano, Mario R Romano

**Affiliations:** ^1^ IRCCS-Fondazione Bietti, Rome, Italy; ^2^ Department of Ophthalmology, Bergamo, Italy; ^3^ Ophthalmology Unit, Department of Medical and Surgical Sciences, University Magna Graecia of Catanzaro, Catanzaro, Italy; ^4^ Catholic University of Milan, Milan, Italy; ^5^ Department of Biomedical Sciences, Humanitas University, Milan, Italy

**Keywords:** diabetic macular edema, dexamethasone, curcumin in hydrophilic carrier, CurcuWIN, central retinal thickness

## Abstract

**Purpose:** This study evaluates if the addition of a curcumin formulation with a polyvinylpyrrolidone-hydrophilic carrier (CHC; Diabec^®^, Alfa Intes, Italy) to intravitreal injections of dexamethasone (DEX-IVT) can affect the morphological retinal characteristics, extending the steroid re-treatment period in patients with diabetic macular edema (DME).

**Methods:** A randomized controlled clinical trial was carried out in DME patients, randomly assigned to receive DEX-IVT or DEX-IVT and a CHC. The evaluation of the mean difference of central retinal thickness (CRT) was the primary aim. Secondary aims were the evaluations of best-corrected visual acuity, differences in the predetermined retinal layer thickness, the number/time of re-treatment, and the assessment of safety.

**Results:** A total of 73 DME patients were included (35 in the control group and 38 in the combined therapy group). In both the control and combined therapy groups, the mean CRT change from T_0_ to the 6 months’ evaluation was significant (*p* = 0.00). The mean CRT result was significantly different at month 4 (*p* = 0.01) between the control and combined therapy groups, with a greater reduction in the combined therapy group, in particular, in patients with ≤10 years of diabetes. A trend of CRT reduction in the combined therapy group has been observed also considering patients with subfoveal neuroretinal detachment. In addition, we observed that the reduction of inner retinal layer thickness was greater in the combination group, in comparison with controls.

**Conclusion:** The combination of a CHC to DEX-IVT is a promising therapeutic option in case of DME, in particular, for patients with early-stage diabetes and with an inflammatory phenotype. Further studies will be necessary to confirm these findings.

## Introduction

Diabetic macular edema (DME) is the leading cause of vision loss in diabetic patients (Daruich et al., 2018).

The treatment of DME still appears difficult. Even if the destruction of the blood–retinal barrier (BRB) is the primary pathological feature, the inflammatory component plays a crucial role in the development of this condition. Consequently, the administration of steroids or anti-vascular endothelial growth factor (anti-VEGF) drugs associated (or not) with laser therapy is a widely used therapeutic approach (Dugel et al., 2015; Berco et al., 2017; Mukkamala et al., 2017). It is worthy of note that anti-VEGF agents and steroids, used in clinical practice, are considerably different in terms of molecular interactions when they bind with VEGF (Platania et al., 2015); therefore, characterization of such features can improve the design of novel drugs in reducing the intravitreal (IVT) injection.

IVT corticosteroids and, among them, slow-release dexamethasone IVT injection (DEX-IVT) have been shown to block the production of several inflammatory mediators, such as VEGF and intercellular adhesion molecule 1 (ICAM-1), and to inhibit leukostasis (Tamura et al., 2005; Platania et al., 2015).

The beneficial effects of DEX-IVT therapy on functional and retinal morphological parameters have been demonstrated in DME (Wang et al., 2008).

To significantly improve the therapeutic approach based on anti-VEGF drugs and steroid IVT injections, the reduction of the administration frequency represents a therapeutic need.

Slow-release IVT implants have been used for this purpose (Regillo et al., 2017), leading to re-treatment times that may vary subjectively from 4 to over 6 months (Scaramuzzi et al., 2015; Pacella et al., 2016; He et al., 2018). To further extend the re-treatment time, some studies are evaluating nutraceutical agents in addition to standard therapies.

In particular, the role of curcumin as an adjuvating therapeutic agent in retinal diseases has been extensively reviewed in the past years (Wang et al., 2013; Riva et al., 2017) and was supported by a recent study on experimental models of diabetic retinopathy (DR) (Li et al., 2016a). Curcumin showed *in vitro* and *in vivo* solid evidence of antioxidant, anti-inflammatory, and antiproliferative activities by suppressing the transcription factor NF-kB (nuclear factor kappa-light-chain-enhancer of activated B cells) activity and thus downregulating the activity of cyclooxygenase-2 (COX-2), nitric oxide synthase (NOS), and others (Muangnoi et al., 2019; López-Malo et al., 2020). Curcumin can also upregulate many factors involved in vessel wall damage and hyperpermeability and can downregulate the expression of pro-inflammatory cytokines (interleukins (ILs) and tumor necrosis factor-α [TNF-α]) and proteins. It also inhibits the VEGF release, which downregulates vascular permeability and retinal neo-angiogenesis (Sarao et al., 2017; Platania et al., 2018).

However, the therapeutic use of curcumin in humans presents some limitations such as poor adsorption, degradation, metabolism, and excretion rates. Consequently, several efforts have been carried out to increase the oral bioavailability of curcumin (Li et al., 2016b).

Among them, the combination of a curcumin formulation (CurcuWIN^®^ Dry Powder 20%) with a polyvinylpyrrolidone-hydrophilic carrier (CHC; Diabec^®^, Alfa Intes, Arpino, Italy) compared with other formulations resulted in its bioavailability in the blood and retina after a single oral administration (Jäger et al., 2014; Sarao et al., 2017; Dei Cas and Ghidoni, 2019).

The efficacy and safety of this formulation for macular edema (ME) of various uncommon etiologies have been recently demonstrated in a retrospective interventional case series, resulting in significant improvement of both functional and anatomical outcomes, with the complete resolution of the edema in most cases (Ferrara et al., 2020).

Based on this background, the aim of the present study is to explore if the addition of a CHC to a DEX-IVT can affect the morphological retinal characteristics, extending the steroid re-treatment period in patients with DME.

## Patients and Methods

### Study Design and Participants

This is a single-blind, randomized controlled clinical trial carried out between February 2018 and March 2020 at three experimental centers in Italy: IRCCS–Fondazione Bietti; Department of Biomedical Sciences, Humanitas Gavazzeni University, Bergamo; and the Department of Ophthalmology of the University of Magna Graecia, Catanzaro. This study was conducted in accordance with the ICH E6 guidelines: “Good Clinical Practice: Consolidated Guidance” and related applicable laws and in accordance with the Declaration of Helsinki.

This study has been approved by the ethics committee of the three experimental centers involved in the study (ClinicalTrials.gov ID: NCT03598205).

The study involved patients with DME in non-proliferative DR, diagnosed by fluorescein angiography and optical coherence tomography (OCT) examination, being treated with DEX-IVT.

Naive patients, patients not treated with anti-VEGF therapy for more than 3 months or with DEX-IVT for more than 6 months, with central retinal thickness (CRT) >300 μm and best-corrected visual acuity (BCVA) evaluated with ETDRS (Early Treatment Diabetic Retinopathy Study) charts at 4 m not <20/400 were included.

Exclusion criteria were considered retinal pathologies other than DME, media opacities limiting the execution and interpretation of diagnostic tests, surgery or para-surgery in the study eye within 3 months prior to the start of treatment, pregnancy, and breastfeeding.

Included patients have been randomly assigned to two groups of treatment:

• Control group: DEX-IVT (0.7 mg)
• Combined therapy group: DEX-IVT (0.7 mg) and 2 tablets/die of CHC. This dosage was reported to be safe and effective in patients with ME, with no reported adverse effects during the follow-up period (Ferrara et al., 2020).

The duration of the study was 6 months.

All patients underwent a comprehensive ophthalmic examination at the baseline (T_0_) and monthly. The ophthalmic examination included the BCVA assessment with ETDRS charts at 4 m, slit lamp biomicroscopy, intraocular pressure measurement, dilated fundus examination, and structural OCT image acquisition with RTVue XR spectral domain (SD)-OCT device (Optovue, Inc., Fremont, CA, USA). This instrument has an A-scan rate of 70,000 scans/s and uses a light source centered at 840 nm and a bandwidth of 45 nm.

Moreover, the thickness of the retinal layers was measured on the structural map, and CRT was analyzed. The OCT software automatically allows measurement of the thickness of individual retinal layers, in particular of the foveal inner retinal layer (IRL) thickness (from the inner limiting membrane to the outer border of the inner plexiform layer) and outer retinal layer thickness, from the inner plexiform layer to Bruch membrane.

DEX-IVT was performed at T_0_ for all included patients. Following the first DEX-IVT, patients were re-treated according to a pro re nata regimen, starting from month 3 if there was a persistence/recurrence of DME, defined as the presence of intraretinal or subretinal fluid on SD-OCT, also in the absence of visual impairment.

### Study Measures

The evaluation of the mean difference of CRT values measured by structural OCT between the two study groups has been considered the primary aim.

Secondary aims were the evaluation of the mean difference of BCVA values, the differences in the predetermined retinal layer thickness detected by structural OCT, the number and the time of re-treatment, and the evaluation of safety.

All measures have been assessed at the baseline (T_0_) and at 1, 3, 4, 5, and 6 months of treatment.

### Statistical Analysis

Descriptive statistics were used to summarize relevant study information. ANOVA for repeated measures followed by *post-hoc* comparisons has been applied to evaluate the experimental results. The statistical software R (R Core Team, 2017), the packages R nlme (Pinheiro and Bates, 2017) and lsmeans (Lenth, 2016) for ANOVA, and *post-hoc* tests have been used. The first- and second-type error thresholds were respectively *α* = 5% and *β* = 20% (i.e., implying a power of 80%).

The primary outcome has been analyzed for the overall population and for the following subgroups: patients with ≤10 years of diabetes and those presenting a subfoveal neuroretinal detachment (SND). The secondary outcomes have been analyzed for the overall population only.

## Results

### Participants

A total of 73 DME patients were included in the study, of whom 35 were randomly assigned to the control group and 38 to the combined therapy group. Baseline characteristics of patients were summarized in Table 1.

**TABLE 1:** Baseline characteristics.

Parameters	Overall	Combined group	Control group
All patients	**n = 73**	**n = 38**	**n = 35**
Age (years), mean ± SD	67 ± 9	66 ± 7	67 ± 10
Diabetes length (years), mean ± SD	13 ± 7	13 ± 7	13 ± 6
Patients with previous IVT, n (%)	38 (52)	20 (52)	18 (51)
Phakic patients, n (%)	43 (58)	24 (63)	19 (66)
Previous laser intervention, n (%)	31 (42)	15 (39)	16 (45)
Patients with ≤10 years diabetes	**n = 30 (41%)**	**n = 14**	**n = 16**
Age (years), mean ± SD	62 ± 8	62 ± 7	61 ± 9
Diabetes length (years), mean ± SD	7 ± 2	7 ± 2	7 ± 2
Patients with previous IVT, n (%)	16 (53)	8 (57)	8 (50)
Phakic patients, n (%)	22 (73)	10 (71)	12 (75)
Previous laser intervention, n (%)	8 (26)	3 (21)	5 (31)
Patients with SND at the baseline	**n = 20 (27%)**	**n = 11**	**n = 9**
Age (years), mean ± SD	69 ± 9	72 ± 6	66 ± 12
Diabetes length (years), mean ± SD	14 ± 7	16 ± 7	13 ± 6
Patients with previous IVT, n (%)	13 (65)	6 (54)	7 (77)
Phakic patients, n (%)	12 (60)	5 (45)	7 (78)
Previous laser intervention, n (%)	8 (40)	5 (45)	3 (34)

Note. IVT, intravitreal; SND, subfoveal neuroretinal detachment.

### Analysis of Central Retinal Thickness Values

#### Overall Population

The CRT values are comparable at the baseline between groups (*p* = 0.07). In both treatment groups, the mean CRT change from T_0_ to the 6 months’ evaluation is significant (*p* = 0.00) and with a decrease in values from 526 ± 108 to 377 ± 155 µm in the control group and from 488 ± 122 to 344 ± 104 µm in the combined therapy group.

**FIGURE 1:**
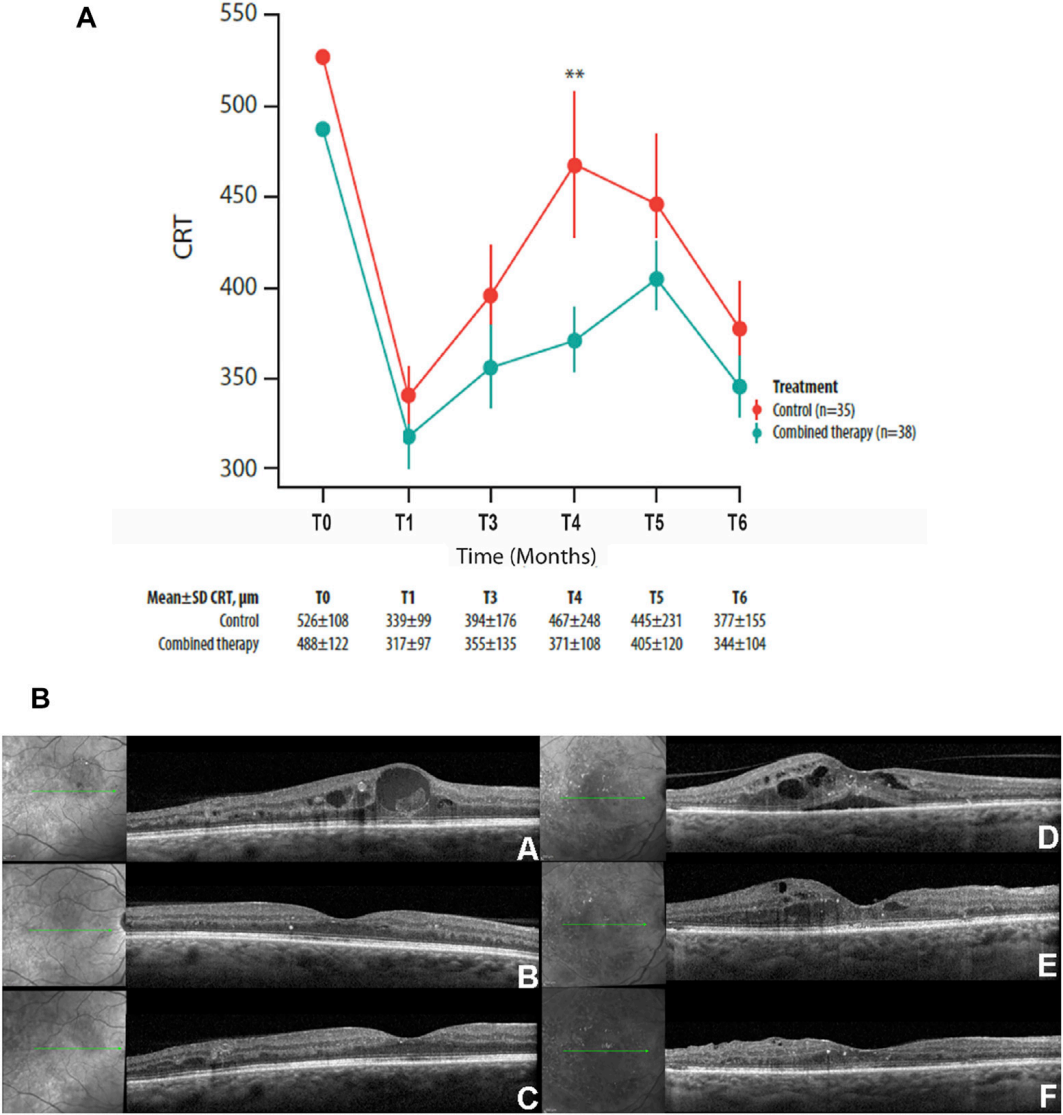
**(A)** Mean ± SD central retinal thickness (CRT) values collected at T_0_ and at each follow-up visit in control and combined therapy groups, considering the overall population. **(B)** Representative OCT scans collected at T_0_ **(A, D)**, at the 4-month follow-up **(B, E)**, and at the 6-month follow-up visits **(C, F)** in combined therapy **(left panel)** and control **(right panel)** patients. In the combined therapy group (left side, A), macular edema, characterized by intraretinal fluid and hyperreflective material inside the cyst, was present at T_0_; macular edema was completely resolved after combined therapy at the 4- and 6-month follow-up **(left side, B** and **C**). In the control group **(right side, D)**, macular edema, characterized by intraretinal and subretinal fluid, was present at T_0_; after DEX-IVT at the 4-month follow-up **(right side, E)**, complete reabsorption of subretinal fluid with persistence of intraretinal fluid was observed; complete reabsorption of intraretinal fluid was recorded at the 6-month follow-up **(right side, F).** **p = 0.01.

The mean CRT values have been compared between the two study groups at the baseline and at each follow-up visit, and the mean CRT result was significantly different at month 4 (*p* = 0.01; Figure 1A), with a greater reduction of the thickness in the combined therapy group in comparison with controls (Figure 1B). Reduction corresponds to 24% in the combined therapy group and to 12% in the control group, compared with baseline values.

#### Patients With ≤10 Years of Diabetes

The mean CRT values of patients with ≤10 years of diabetes have been compared between the two treatment groups, with the aim of verifying whether the adjuvant effect of the CHC therapy was more evident in the early stage of diabetes.

**FIGURE 2:**
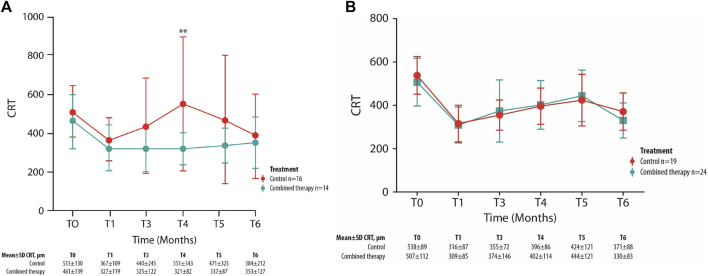
Mean ± SD central retinal thickness (CRT) values collected at T_0_ and up to the 6-month follow-up visit in control and combined therapy groups, considering patients with ≤10 years of diabetes **(A)** or >10 years of diabetes **(B)**. **p = 0.002.

The statistical analysis indicated a significant difference in the mean CRT values between groups at month 4 (*p* = 0.002) (Figure 2A).

These data are strengthened by the lack of significance found in the same analysis carried out on patients with diabetes for >10 years (n = 43) (Figure 2B).

#### Patients With Subfoveal Neuroretinal Detachment

**FIGURE 3:**
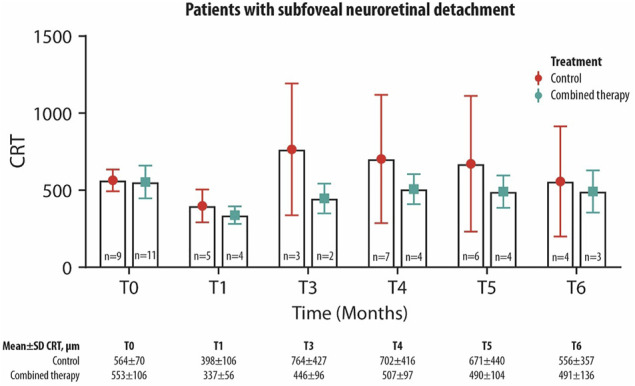
Mean ± SD central retinal thickness (CRT) values collected at T_0_ and up to the 6-month follow-up visit in control and combined therapy groups, considering patients with SND.

The mean CRT values have been compared between the two treatments group considering only patients who present SND at the baseline and for each follow-up visit. A trend of greater reduction of CRT values can be observed in patients treated with combined therapy compared with those in the control group (Figure 3), despite not being statistically significant. At each follow-up visit, the mean number of patients with an SND was 17% (n = 6) of total patients in the control group and 10% (n = 4) in the combined therapy group.

### Secondary Outcomes

#### Best-Corrected Visual Acuity Analysis

The BCVA values are non-homogeneous at the baseline between groups (*p* = 0.00).

The comparison between the mean BCVA values at each follow-up between the two study groups did not show any significant difference (baseline BCVA = 50.0 ± 18.7 in the control group and 48.5 ± 17.7 in combined therapy group; 6 months BCVA = 54.8 ± 17.0 in the control group and 48.9 ± 19.8 in combined therapy group).

#### Anatomical Findings on Optical Coherence Tomography

**FIGURE 4:**
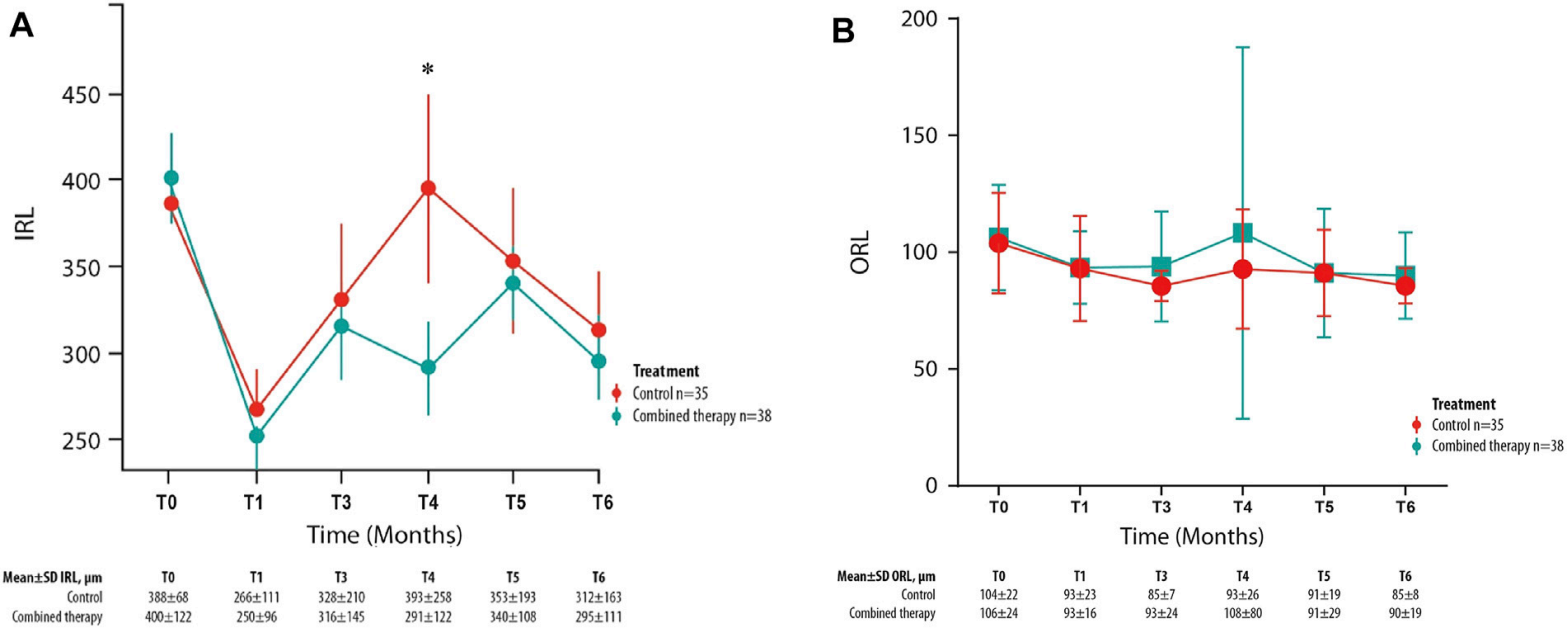
Mean ± SD inner retinal layer (IRL) **(A)** and outer retinal layer **(B)** values collected at T_0_ and for each follow-up visit in control and combined therapy groups. *p = 0.045.

Among the different retinal layer thickness measured by structural OCT, a significant difference between the two study groups was detected in IRL thickness values with a significantly greater reduction at month 4 in the combined group in comparison with the control group (Figure 4A). Compared with T_0_ values, at T4, the IRL thickness variation is equal to −28% in the combined therapy group and +1% in the control group.

At baseline, this parameter was homogeneous between groups (*p* = 0.78).

Otherwise, the mean outer retinal layer thickness values were not significantly different between the two study groups in any follow-up visit (Figure 4B).

#### Comparison Between Dexamethasone Intravitreal Injection Number and Reinjection Time

During the study period, there was no significant difference in the mean number of treatments (1.4 ± 0.5 in the control group and 1.6 ± 0.5 in the combined treatment group) nor a significant difference in the re-injection mean time (4.5 ± 0.8 months in the control group, 4.6 ± 0.5 months in the combined therapy group) between the two study groups.

During the study, 31 patients out of 73 (42%) were treated with only one DEX-IVT: 15 in the control group and 16 in the combined therapy. Otherwise, most of the patients were re-treated (n = 42, 58%): two control patients within the first 3 months, the others after the 4 months’ follow-up, 19 in the control group, and 21 in the combined treatment.

#### Safety Assessment

No events of hypertonia, endophthalmitis, or retinal detachment were reported by patients during the study period.

## Discussion

In this study, we aimed to verify if the addition of a CHC to a DEX-IVT in DME patients, compared with DEX-IVT alone, can affect the morphological retinal response at 6 months.

The efficacy of DEX-IVT therapy in DME on functional and retinal morphological parameters has been widely demonstrated (Wang et al., 2008), by exerting specific effects on the inflammatory component in DME. To further extend the re-treatment time, some studies are evaluating nutraceutical agents in addition to standard therapies.

In particular, emerging evidence of the pharmacological effects of curcumin led to this compound being considered as a potentially beneficial treatment of various retinal diseases, including those complicated by DME (Peddada et al., 2019). DME is recognized as a neurovascular complication of DR, and the involvement of inflammatory processes in this pathology particularly characterizes the early stages of DR (Rossino et al., 2019).

Curcumin shows anti-inflammatory, antioxidant, antiangiogenic, and neuronal- and vascular-protective properties due to its capability to target and regulate multiple signaling pathways (Rossino et al., 2019). An inhibitory effect has also been documented of curcumin on cyclooxygenase, NF-κB, TNF-α, IL-1, IL-6, IL-8, and free radical production.

Moreover, curcumin shows an inhibitory activity on lipoteichoic acid-activated microglial cells. Neuroinflammation plays a key role in the pathogenesis of DR, where the microglia become activated, producing inflammatory mediators (Malchiodi-Albedi et al., 2008; Abcouwer, 2013; Farajipour et al., 2018).

However, the clinical use of curcumin has been limited by its pharmacokinetics, being poorly soluble and rapidly metabolized and eliminated (Yu et al., 2018).

To increase the oral bioavailability of curcumin, a CHC has been conjugated (CHC, Diabec^®^). This combination was demonstrated to be bioavailable in the human blood with a concentration that is 46 times higher than that of other curcumins formulations and reached the retinal target after a single administration in a rabbit model (Sarao et al., 2017; Dei Cas and Ghidoni, 2019).

The results of the study show a significant reduction of the mean CRT values in both groups after treatment. In addition, a significant CRT reduction has been observed in the combined therapy group at month 4 in comparison with controls, concomitant with the reduction of the dexamethasone effect, administered by DEX-IVT at the baseline to all patients.

Of note, the reduction of the mean CRT values at month 4 is more evident in the combined therapy group if we consider patients with early-stage diabetes (<10 years of diabetes duration) or patients who present an SND.

In addition, we observed that the reduction of intra/subretinal fluid was greater in the combination group and more evident in the IRL.

These results could be related to the complex properties of a CHC that exert a synergic anti-inflammatory effect with DEX-IVT and by reducing the glia activation in the inner retina.

To better understand this result, we can speculate about its correlation with the pathogenesis of DME in the early stages. The BRB breakdown is a typical event in early-stage DR, and the underlying mechanisms causing this vascular dysfunction result in the increased vascular permeability and degeneration of retinal capillaries (Anand et al., 2007).

The breakdown of both the inner BRB (iBRB) and outer BRB triggers the development of DME at any stage of DR (Shin et al., 2014). In particular, the breakdown of the iBRB especially is a hallmark of DME (Das, 2016).

The iBRB is composed of the endothelium cells on the basal lamina, enveloped by the processes of Müller cells and pericytes, which are responsible for the activity of retinal endothelial cells transmitting regulatory signals (Vinores, 2010) in the inner retina.

Several cytokines and growth factors are responsible for BRB breakdown in the early stages through multiple signaling pathways, leading to the loss of adherents and tight-junction proteins between endothelial cells, responsible for the regulation of vascular permeability (Hosoya and Tachikawa, 2012).

This suggests that the adjuvant efficacy of a CHC is enhanced especially in the earliest stages of the retinal pathology when the structural damage is still limited and the vascular network is not completely compromised.

In addition, our results showed a trend of greater reduction of CRT values among patients with SND treated with combined therapy as compared with those in the control group, with the smallest percentage of SND in the combined therapy at each follow-up visit. DME with SND has been considered as a distinct DME pattern associated with a major ocular inflammatory condition, including higher levels of IL-6 in the vitreous and increased number of hyperreflective retinal spots, considered as signs of activated microglial cells in the retina. These results corroborate a possible relevant role of curcumin on the inflammation process mitigation.

In our population, no differences in the mean number of IVT and in the re-treatment time have been observed. This could be related to the defined study period of 6 months, and a longer one could allow a better evaluation of this parameter. In addition, the re-treatment was performed by evaluating not only the reduction of intra/subretinal fluid related to the CRT parameter but also the BCVA, a functional parameter that often does not correlate with the anatomical variations (Förster, 2008; Browning and Fraser, 2008).

This study presents some limitations such as the small size of the sample, the short duration of the follow-up, and the inclusion of naïve and previously treated patients. In addition, not considering the metabolic parameters as influencing factors for the morphological and functional outcomes could be another limitation.

## Conclusion

The oral administration of a CHC in addition to DEX-IVT in patients affected by DME is well-tolerated and resulted in a greater improvement of morphological findings (significant reduction of CRT and IRL values), suggesting a major effect on DME. The beneficial effect of the additional treatment with a CHC is even more evident when considering the subpopulation of patients with ≤10 years of diabetes or who present an SND.

In conclusion, the combination of a CHC to DEX-IVT has promising results as a therapeutic option in case of DME, in particular for patients with early-stage diabetes and with an inflammatory phenotype. Further studies will be necessary to validate this therapeutic strategy and confirm our findings.

The treatment of DME still appears difficult. Considering the solid *in vitro* and *in vivo* evidence about the antioxidant, anti-inflammatory, and antiproliferative activities of curcumin, its potential as an adjuvating therapeutic agent in retinal diseases has been extensively reviewed, and a recent study on experimental models of DR supported this hypothesis. This study demonstrates that the oral administration of a curcumin formulation (CurcuWINÂ^®^ Dry Powder 20%) with a CHC (DiabecÂ^®^, Alfa Intes, Italy) in addition to slow-release DEX-IVT in DME patients is well-tolerated and resulted in a greater improvement of morphological findings (significant reduction of CRT and IRL), suggesting a major effect on DME. The beneficial effect of the additional treatment with a CHC was more evident when considering the subpopulation of patients with ≤10 years of diabetes or who present an inflammatory phenotype, further increasing our knowledge about this therapeutic approach.

## Data Availability

The raw data supporting the conclusions of this article will be made available by the authors, without undue reservation.
